# Hybrid Fourier-domain mode-locked laser for ultra-wideband linearly chirped microwave waveform generation

**DOI:** 10.1038/s41467-020-17264-8

**Published:** 2020-07-30

**Authors:** Jian Tang, Beibei Zhu, Weifeng Zhang, Ming Li, Shilong Pan, Jianping Yao

**Affiliations:** 1 Microwave Photonics Research Laboratory, School of Electrical Engineering and Computer Science, University of Ottawa, 25 Templeton Street, Ottawa, Ontario K1N6N5 Canada; 2 State Key Laboratory on Integrated Optoelectronics, Institute of Semiconductors, Chinese Academy of Sciences, Beijing, 100083 China; 3 School of Electronic, Electrical and Communication Engineering, University of Chinese Academy of Sciences, Beijing, 100049 China; 4 College of Electronic and Information Engineering, Nanjing University of Aeronautics and Astronautics, Nanjing, 210016 China

**Keywords:** Electrical and electronic engineering, Microwave photonics

## Abstract

We show the generation of a tunable linearly chirped microwave waveform (LCMW) with an ultra-large time-bandwidth product (TBWP) based on a hybrid Fourier-domain mode-locked (FDML) laser. The key device in the hybrid FDML laser is a silicon photonic integrated micro-disk resonator (MDR) which functions as an optical bandpass filter, to have strong wavelength selectivity and fast frequency tunability. By incorporating the integrated MDR in the fiber-based ring cavity to perform frequency-domain mode locking, an FDML laser is realized and a broadband frequency-chirped optical pulse is generated. By beating the frequency-chirped optical pulse with an optical carrier from a laser diode (LD) at a photodetector (PD), an LCMW is generated. The bandwidth of the LCMW is over 50 GHz and the temporal duration is over 30 µs, with an ultra-large TBWP of 1.5 × 10^6^. Thanks to the strong tunability of the MDR in the FDML laser, the generated LCMW is fully tunable in terms of bandwidth, temporal duration, chirp rate, and center frequency.

## Abstract (web summary)

Linearly chirped microwave waveforms with high time-bandwidth products would have important applications but are difficult to achieve. Here the authors generate such a waveform in a photonic setting by implementing a micro-disk resonator as a tunable optical bandpass filter in a Fourier-domain mode-locked laser.

## Introduction

Thanks to its wide applications such as aerial photography^1^, detection^2^, and surveillance^3^, unmanned aerial vehicle (UAV) technology has enjoyed rapid development in recent years. Since UAVs usually have a small size in the order of a few meters or smaller, it is very challenging for current radar systems to detect and track UAVs^4,5^. For example, to quickly and clearly capture a UAV with a size of one square meter, a microwave imaging system is required to have a resolution as high as a few centimeters^5–7^. However, most of the current microwave imaging systems usually have a resolution of a few meters^8–10^. Therefore, there is a significant need to develop high-resolution microwave imaging systems to detect and track UAVs. To solve this problem, pulse compression is an enabling technique, in which a linearly chirped microwave waveform (LCMW) with a very large time-bandwidth product (TBWP) is needed^11^. In particular, an LCMW with a large TBWP in the order of 10^4^ or more is highly preferred for UAV detection in a microwave imaging system^12^. Conventionally, an LCMW is generated using a digital frequency synthesizer^13^. Due to the limited speed of currently available electronic circuits, an LCMW generated using the digital approach is limited in the center frequency and bandwidth, which significantly limits the imaging resolution^14,15^.

To generate an LCMW with a large TBWP, a solution is to use modern optical technology, which has the key advantages including high frequency and broad bandwidth^16^. In the past few years, different photonics approaches have been proposed and demonstrated for LCMW generation, including direct space-to-time (STM) pulse shaping^17–19^, spectral shaping and wavelength-to-time (SS-WTT) mapping^20–23^, optical heterodyne detection^24–26^ and Fourier-domain mode-locked (FDML) optoelectronic oscillation (OEO)^27^. Among these approaches, the STM technique is the one that can be easily implemented. However, due to the limited number of channels, the generated LCMW usually has a TBWP as small as ~50^19^. To increase the TBWP, the approach based on SS-WTT mapping has been proposed. The main problem with this approach is that a dispersive element with large dispersion is required to realize WTT mapping, to generate an LCMW with a large TBWP, which is difficult to achieve or a long and heavy dispersive fiber is needed^20,21,28^. To avoid using a dispersive element with large dispersion, one solution is to beat two optical signals from a continuous wave (CW) laser source and a frequency-swept laser source. The main limitation at this method is that the generated LCMW has a high phase noise, due to the fact that the two laser sources are not phase correlated^25^. To overcome this problem, heterodyne beating of two optical combs from the same laser source has been proposed, in which an LCMW with an ultra-large TBWP as large as 3 × 10^8^ was experimentally generated with a low phase noise^26^. However, this approach needs two independent microwave sources including a fast frequency-sweeping microwave source, which makes the system bulky and costly. To avoid using microwave sources, an FDML OEO has been experimentally demonstrated for LCMW generation, in which an LCMW having a TBWP of 1.6 × 10^5^ was generated^27^. However, the TBWP is still small, which is limited by the frequency-tunable range of the microwave photonic filter in the OEO loop, to achieve frequency-domain mode locking.

In this paper, we propose and experimentally demonstrate an approach to generate a LCMW with an ultra-large TBWP based on a hybrid FDML laser incorporating a silicon photonic integrated tunable micro-disk resonator (MDR). Similar to a regular fiber ring laser, the hybrid FDML laser has a fiber ring structure, but a fast and frequency-tunable silicon photonic integrated MDR is incorporated in the ring cavity to achieve Fourier-domain mode locking^29–31^. In our proposed configuration, the silicon photonic integrated MDR is designed to have a top-placed metallic micro-heater which is used to perform narrow-band wavelength selection and fast wavelength tuning. By applying a driving signal with a frequency that is a multiple of the free spectral range (FSR) of the ring cavity to the MDR, Fourier-domain mode locking is achieved and a frequency-chirped optical pulse is generated. In our experimental demonstration, the frequency-chirped optical pulse has a maximum bandwidth of 0.8 nm and from a maximum temporal width of 30 µs. By beating the generated frequency-chirped optical pulse with a CW optical carrier from a laser diode (LD) at a high-speed photodetector (PD), an LCMW with a bandwidth over 50 GHz and a temporal duration over 30 µs, corresponding to an ultra-large TBWP of 1.5 × 10^6^, is generated. Thanks to the strong tunability of the photonic integrated MDR in the FDML laser, the generated LCMW is fully tunable in terms of bandwidth, temporal duration, chirp rate, and center frequency. The implementation of such a hybrid FDML laser with a silicon photonic integrated MDR for highly tunable LCMW generation with an ultra-large TBWP paves the way toward practical applications of FDML lasers for generating large TBWP microwave waveforms for high-resolution microwave imaging and sensing.

## Results

### Hybrid FDML laser

**Fig. 1:**
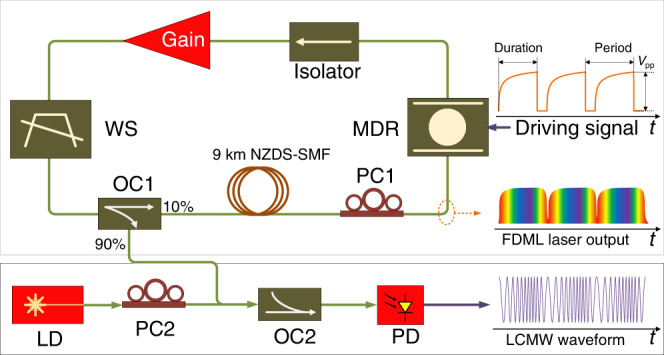
Schematic diagram of the proposed LCMW generation system. WS wave-shaper, OC optical coupler, PC polarization controller, LD laser diode, NZDS-SMF none-zero dispersion-shifted single mode fiber, PD photodetector.

A hybrid FDML laser is realized based on a fiber ring cavity in which a tunable optical filter is incorporated to perform Fourier-domain mode locking. Figure 1 shows the schematic of the proposed hybrid FDML laser, which has a discrete fiber-based ring cavity incorporating an integrated silicon-based tunable MDR filter having a top-placed metallic micro-heater for thermal tuning. By applying a driving signal with a frequency that is a multiple of the FSR of the ring cavity to the micro-heater on top of the MDR, Fourier-domain mode locking is achieved and a broadband frequency-chirped optical pulse is generated. By controlling the temporal profile of the driving signal, the generated optical pulse is linearly frequency chirped. Specifically, when the driving signal has a parabolic profile, the generated optical pulse is frequency-chirped with linearly increasing frequency, as shown in Fig. 1. Then, the generated linearly frequency-chirped optical pulse is sent to a high-speed PD together with an optical carrier from an LD, to generate an LCMW due to heterodyne beating at the PD. Since the linearly frequency-chirped optical pulse has a broad bandwidth and wide temporal duration, the generated LCMW has an ultra-large TBWP. In addition, when the driving signal applied to the MDR is tuned, the frequency-chirped optical pulse from the FDML laser can be tuned in terms of bandwidth, temporal duration, chirp rate, and center frequency, leading to the generation of a fully tunable LCMW.

**Fig. 2:**
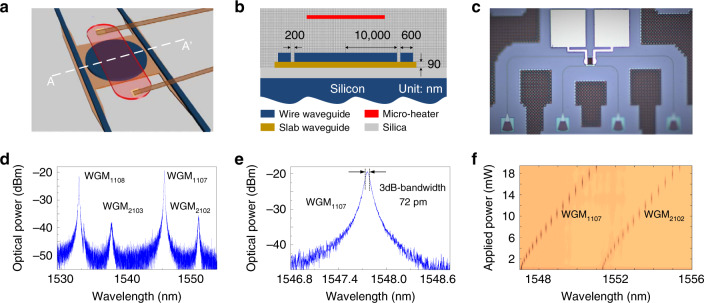
Design of the microdisk resonator (MDR). **a** Perspective view of the thermally tunable MDR. **b** Cross-sectional view of the MDR along the dashed line AA’ shown in (**a**). **c** Micrograph of the fabricated MDR. **d** Spectral response of the MDR from the drop port. **e** Zoom-in view of the WGM_1,107_, the 3-dB bandwidth is 72 pm. **f** Spectrum tuning of the MDR by applying a voltage to the micro-heater.

In the implementation of the FDML laser, mode selection is realized using a highly frequency-selective and fast frequency-tunable optical filter. The higher the frequency selectivity of the optical filter, the more precise the mode selection. To enable Fourier-domain mode locking, the repetition time of the driving signal applied to the optical filter needs to match the round-trip time of the laser ring cavity^29,31,32^. In the proposed FDML laser, a specially designed add-drop MDR is employed as the optical filter. The MDR is designed to have an additional slab waveguide to wrap the disk and bus waveguide, to increase the wavelength selectivity and to enhance the optical coupling between the bus waveguide and the disk. Figure 2a gives the perspective view of the MDR. A slab waveguide is added to wrap the disk and the bus waveguide to weaken the disk sidewall roughness, and thus increase the confinement of the optical field^33,34^. Figure 2b shows the cross-sectional view of the MDR along the dashed line AA’, shown in Fig. 2a. The disk has a radius of 10 μm and the coupling gap between the bus waveguide and the disk is 200 nm. The chip is fabricated with the use of 193 nm lithography fabrication process. Figure 2c shows the image of the chip prototype captured by a microscope camera. Figure 2d shows the measured transmission spectrum at the drop port of the MDR. The first- and second-order whispering gallery modes (WGMs) are effectively excited, in which the first-order WGMs have an FSR of 10.56 nm, and the second-order WGMs have an FSR of 10.76 nm. Figure 2e is a zoom-in view of the WGM_1,107_, which has a -3-dB bandwidth of 72 pm, corresponding to a Q-factor as large as 2.1 × 10^4^. By applying a driving signal to the micro-heater on top of the MDR, the filter is thermally tuned. Figure 2f shows the wavelength tuning of the MDR. With the increase in the applied electrical power, the WGM_1,107_ and WGM_2,102_ are red shifted. For the WGM_1,107_, the wavelength shift rate is calculated to be 155 pm/mW, and for the WGM_2,102_, the wavelength shift rate is calculated to be 196 pm/mW. In addition, thanks to the strong thermal-optic effect in silicon, the response time is in the order of tens of microseconds, which is much faster than a conventional tunable optical filter such as liquid crystal based tunable optical filter^35^, and acoustically tunable optical filter^36^, a feature that is highly needed for Fourier-domain mode locking.

### Frequency-chirped optical pulse generation

**Fig. 3:**
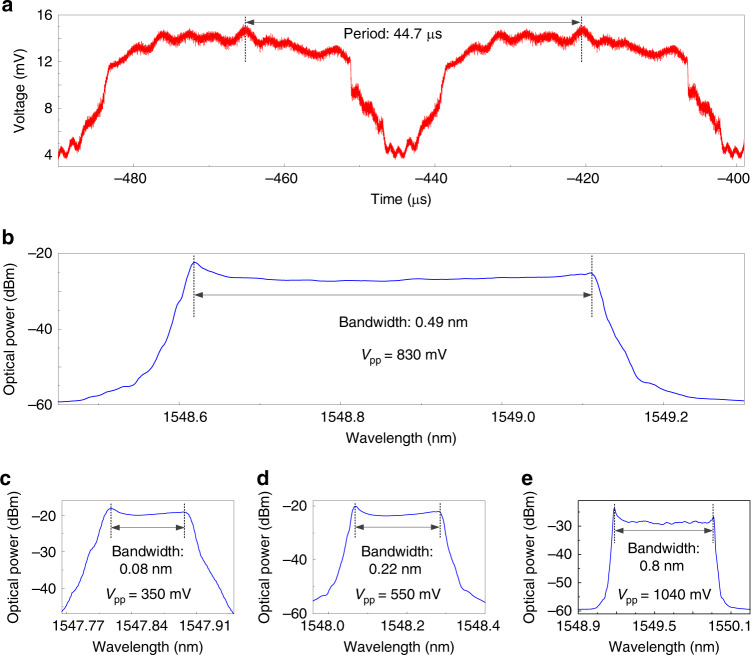
Frequency-chirped optical pulse generated by the Fourier-domain mode-locked (FDML) laser. **a** Temporal profile and **b** optical spectrum of the frequency-chirped optical pulse from the FDML laser when the bias voltage *V*_PP_ applied to the micro-heater is 830 mV. The optical spectrum of the frequency-chirped optical pulse from the FDML when the bias voltages *V*_PP_ applied to the micro-heater are **c** 350 mV, **d** 550 mV, and **e** 1040 mV.

First, an experiment is performed to demonstrate the operation of the hybrid FDML laser with the use of the silicon photonic integrated MDR chip. Since the length of the laser cavity is around 9 km, a round-trip time is calculated to be about 44.4 µs. In the experiment, a driving signal with a repetition time of 44.7 µs having a parabolic profile is applied to the micro-heater. The tuning of the driving signal has a resolution of 1 mHz, to precisely tune the repetition time to make it match precisely the cavity round-trip time to ensure a stable operation of the FDML laser. The generated frequency-chirped optical pulse is measured by an optical spectrum analyzer (OSA) (see Supplementary Note 1). Figure 3a shows the temporal profile of the frequency-chirped optical pulse after photodetection at a high-speed PD, which is captured by a real-time oscilloscope. As can be seen, the waveform is periodic with a repetition time of 44.7 µs, which is identical to the repetition time of the driving signal applied to the MDR. Figure 3b shows the optical spectrum of the generated frequency-chirped optical pulse, measured using an OSA. The pulse has a 3-dB bandwidth as large as 0.49 nm. Such a broad bandwidth is of significant benefit to the generation of a wideband LCMW based on heterodyne beating. When the driving signal is tuned, the generated optical pulse is also tuned. Figure 3c–e shows the tuning of the frequency-chirped optical pulse. As can be seen, with the peak-to-peak voltage (*V*pp) of the driving signal applied to the micro-heater is increased from 350 to 1040 mV, the bandwidth of the frequency-chirped optical pulse is increased from 0.08 to 0.8 nm. Thanks to the broad bandwidth of the frequency-chirped optical pulse, an ultra-large TBWP is generated when the optical pulse is heterodyne beating with an optical carrier from an LD.

### LCMW generation

**Fig. 4:**
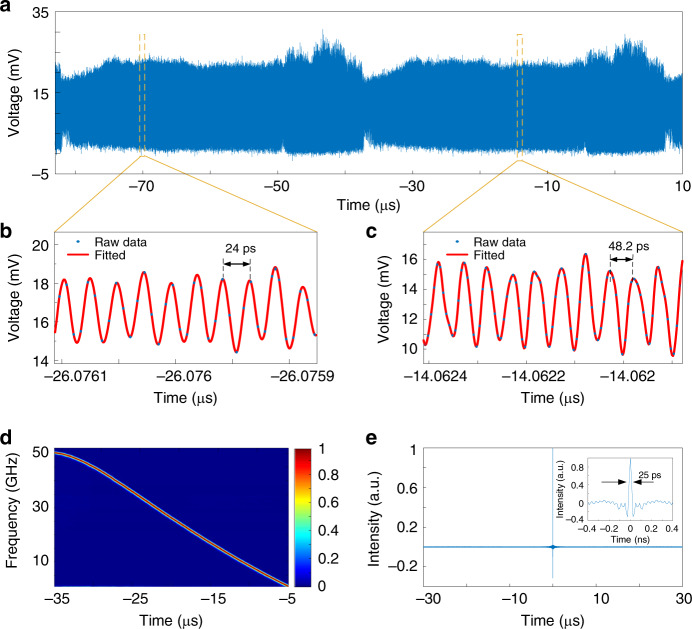
Generated linearly chirped microwave waveform (LCMW). **a** Temporal waveform of the generated LCMW with a positive chirp rate by beating the frequency-chirped optical pulse with an optical carrier from an LD at a high-speed PD. The zoom-in views of the waveform are shown in (**b** and **c**). The blue dots in (**c**) are the originally recorded data and the red lines are the fitted waveforms. **d** Spectrogram of the waveform shown in (**a**). **e** Compressed pulse by autocorrelation.

**Fig. 5:**
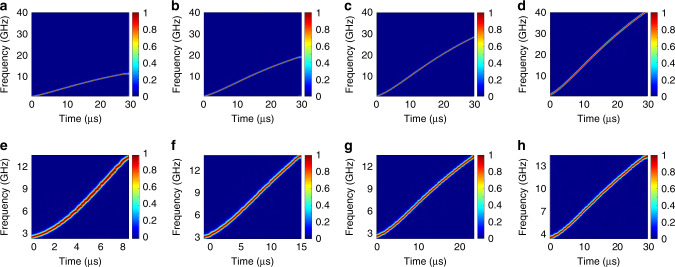
Tuning of the generated LCMW with a tunable bandwidth. The bandwidth is **a** 10.2 GHz, **b** 20.1 GHz, **c** 27.9 GHz, and **d** 38.1 GHz, while maintaining the pulse duration at 30 µs. Tuning of the generated LCMW with a duration of **e** 8.5 µs, **f** 16.4 µs, **g** 22.2 µs, and **h** 30 µs, while maintaining the pulse bandwidth around 11 GHz.

Then, the generated frequency-chirped optical pulse is combined with a CW optical carrier from an LD and beat at a high-speed PD. At the output of the PD, an LCMW is generated. Figure 4a shows the temporal waveform of the generated LCMW when the frequency-chirped optical pulse with a bandwidth of 0.4 nm from the FDML laser is used. Figure 4b and c gives the zoom-in views of the generated LCMWs at different time locations. As can be seen, the instantaneous frequency of the waveform is changing with time, which confirms the effectiveness of the LCMW generation with the use of the FDML laser. Figure 4d presents its spectrogram of the generated LCMW which exhibits a linear chirp profile. As can be seen, the generated LCMW has a time duration of 30 μs and a bandwidth of 50 GHz. From the time-domain waveform and its carrier frequency distribution, the TBWP of the generated LCMW is calculated to be as large as 1.5 × 10^6^. To evaluate the linearity of the generated LCMW, the instantaneous frequency is linearly fitted to an ideal LCMW. An *R*-square value is calculated which is 0.99763, a very high value confirming that the waveform has good linearity. A slight nonlinearity of the frequency distribution of the generated LCMW that can be observed from Fig. 4d is mainly resulted from the nonlinear response of the MDR to the driving signal, which can be reduced or completely eliminated if the driving signal is pre-distorted to fully compensate for the nonlinearity. Figure 4e shows a compressed pulse by calculating the autocorrelation. The temporal width of the compressed pulse is 25 ps. By comparing the pulse widths of the waveforms in Figs. 4a and e, a pulse compression ratio as large as 1.2 × 10^6^ is obtained. Note that when calculating the autocorrelation, the direct-current (DC) component in the generated LCMW is removed before the calculation. By tuning the *V*pp and the parabolic profile of the driving signal applied to the micro-heater, the frequency-chirped optical pulse can be tuned in terms of bandwidth, temporal duration, chirp rate, and repetition rate. When the frequency-chirped optical pulse is combined with an optical carrier and beat at a high-speed PD, a tunable LCMW is generated. Specifically, the parameters of the generated LCMW, in terms of bandwidth, duration time and repetition rate, can be tuned by tuning the driving signal through tuning the *V*pp, the temporal duration, and the repetition time, respectively (the time-domain profile of the driving signal can be found in Supplementary Note 2). Figure 5a–d shows the spectrograms of the generated LCMW with different bandwidths while maintaining an identical temporal duration by controlling the *V*pp of the driving signal. As can be seen, the bandwidth is increased from 10.2 to 38.1 GHz when the *V*pp of the driving signal is increased from 350 to 650 mV. The chirp rate is correspondingly increased from 0.34 to 1.27 GHz/µs. By using a PD with a wider bandwidth, the bandwidth of the generated LCWM could be largely increased, and the chirped rate could also be increased accordingly. The *R*-square value of the spectrogram in Fig. 5a is relatively smaller than those in Fig. 5b–d which indicates that a lower *V*pp will lead to a poorer linearity of the LCMW. Figure 5e–h shows the spectrograms of the generated LCMWs with an identical bandwidth but different temporal durations by controlling the parabolic duration of the driving signal. The duration is increased from 8.5 to 30.0 µs when the parabolic duration of the driving signal is increased from 8.5 to 30.0 µs, and the chirp rate of the generated LCWM is tuned from 1.24 G to 0.37 GHz/µs. The *R*-square value of the spectrogram of Fig. 5e is relatively smaller than those in Fig. 5f–h, which indicates that a shorter driving duration could lead to poorer linearity of the LCMW. Table 1 summarizes the parameters of the generated LCMWs.

**Table 1:** Summary of the parameters of the generated LCMWs in Fig. 5.

Parameter	a	b	c	d	e	f	g	h
Bandwidth (GHz)	10.2	20.1	27.9	38.1	10.5	10.8	10.9	11.0
Duration (μs)	30	30	30	30	8.5	16.4	22.2	30
Chirp rate (GHz/μs)	0.3	0.67	0.93	1.27	1.24	0.67	0.49	0.37
*R*-square	0.99665	0.99807	0.99805	0.998	0.98834	0.99511	0.99857	0.99892

**Fig. 6:**
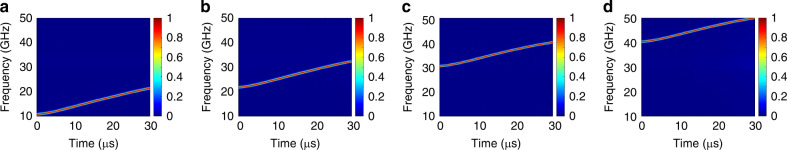
Center frequency tuning of the generated LCMW. **a** 16.0 GHz, **b** 26.6 GHz, **c** 37.0 GHz, and **d** 45.5 GHz. During tuning the pulse bandwidth and pulse duration are maintained to be 10 GHz and 30 µs.

The center frequency of the generated microwave waveform is determined by the frequency difference between the frequency-chirped optical pulse and the CW optical carrier. In our experiment, by changing the wavelength of the CW optical carrier, the center frequency of the generated LCMW is tuned. Figure 6a–d shows the spectrograms of the generated LCMWs with different center frequencies. As can be seen the center frequency is increased from 15.68 to 44.73 GHz when the wavelength of the CW optical carrier is reduced from 1547.89 to 1547.65 nm. If a PD with a wider bandwidth is employed, the center frequency of the generated LCMW can be significantly higher. Since the parabolic driving signal is maintained unchanged, the chirp rates of the LCMWs are maintained unchanged. The *R*-square values are kept unchanged which indicates the linearity of the LCMWs is maintained without change when tuning the center frequency of the LCMW. Table 2 summarizes the parameters of the generated LCMWs.

**Table 2:** Summary of the parameters of the LCMW in Fig. 6.

Parameter	a	b	c	d
Bandwidth (GHz)	10.56	10.50	10.48	10.48
Duration (μs)	30	30	30	30
Chirp rate (GHz/μs)	0.35	0.35	0.35	0.35
Center frequency (GHz)	15.68	26.63	37.07	44.73
*R*-square	0.99872	0.99866	0.99754	0.99755

**Fig. 7:**
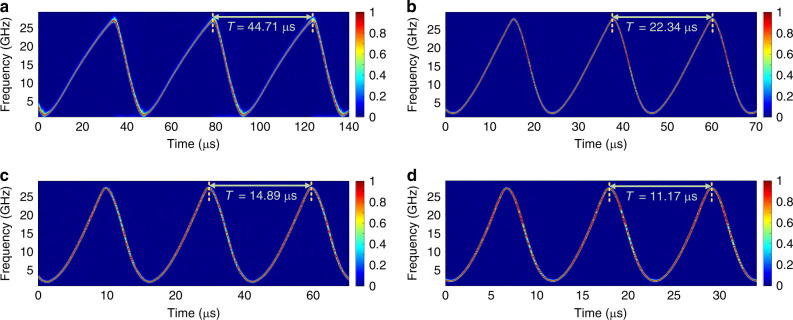
Spectrogram of the generated LCMW with different repetition time. **a** The fundamental repetition time of 44.71 µs with the *V*_PP_ of the driving signal applied on the MDR is 469 mV. The generated LCMW with the second, third and fourth harmonic repetition time are shown in (**b**, **c** and **d**), the corresponding *V*_PP_ applied on the MDR are 684, 910, and 1140 mV, respectively.

The repetition rate of the frequency-chirped optical pulse can be tuned by adjusting the repetition time of the parabolic driving signal. To ensure effective Fourier-domain mode locking, the repetition rate should be controlled to be equal exactly to a multiple of the FSR of the ring cavity. Figure 7a–d shows the spectrograms of the generated LCMWs with different repetition times. In the experiment, the repetition time is reduced from 44.71 to 11.17 µs. To maintain the bandwidth of the LCMW fixed at around 25 GHz, the *V*pp of the driving signal is increased from 469 to 1140 mV. The reason that an increased *V*_PP_ is needed to maintain a fixed bandwidth is because the average electrical power applied to the MDR is decreased when the repetition time of the driving signal is reduced. The shortest repetition time is determined by the highest *V*_PP_ of the driving signal that will not damage the thermal resistance in the MDR (as shown in Fig. 2c). The *R*-square value is decreased from 0.99714 to 0.988 when the repetition time is decreased from 44.71 to 11.17 µs, which can be improved by fine tuning the profile of the driving signal to compensate for the nonlinearity. Table 3 summarizes the parameters of the generated LCMWs.

**Table 3:** Summary of the parameters of the LCMW in Fig. 7.

Parameter	a	b	c	d
Bandwidth (GHz)	24.73	24.49	24.73	24.68
Duration (μs)	29.58	13.14	8.46	6.24
Chirp rate (GHz/μs)	0.83	1.86	2.92	3.95
Repetation time (μs)	44.71	22.34	14.89	11.17
*R*-square	0.99714	0.99725	0.9907	0.988

Note that due to the optical carrier from the LD used to perform heterodyne beating is independent from the optical pulse from the FDML, the phase noise of the optical carrier will be translated to the generated LCMW. A potential solution to make the optical carrier from the LD be phase correlated with the optical pulse from the FDML laser is to select one longitudinal mode from the FDML to phase lock the LD, to make the two wavelengths highly coherent. Thus, the phase noise could be significantly reduced^37^.

## Discussion

We have proposed and experimentally demonstrated an approach to generate a tunable LCMW with an ultra-large TBWP, which was realized based on a hybrid FDML laser. The key device in the hybrid FDML laser was the silicon photonic integrated MDR which was specially designed to make it have a narrow bandwidth and fast tunability needed for Fourier-domain mode locking. By tuning the silicon photonic integrated MDR to which a driving signal that has a repetition rate that is a multiple of the FSR of the ring cavity was applied, Fourier-domain mode locking was achieved. In the experiment, a frequency-chirped optical pulse with a bandwidth up to 0.8 nm and a temporal width up to 30 µs was generated. By heterodyne beating the generated frequency-chirped optical pulse with a CW optical carrier from an LD at a high-speed PD, an LCMW was generated. The bandwidth of the LCMW was over 50 GHz and the temporal duration was over 30 µs. The corresponding TBWP was over 1.5 × 10^6^. By pulse compression, a significantly compressed pulse with a compression ratio as large as 1.2 × 10^6^ was achieved. In addition, thanks to the strong tunability of the MDR, the generated LCMW could be fully tunable in terms of bandwidth, temporal duration, chirp rate, and center frequency. This is the first time, to the best of our knowledge, that a hybrid FDML laser employing a silicon photonic integrated MDR as a tunable filter to generate an LCMW having an ultra-large TBWP with a full tunability was demonstrated. The successful demonstration of such a system represents a significant step towards ultra-wideband and arbitrarily tunable LCMW generation which can find important applications in high-resolution radar and microwave imaging systems.

## Methods

### Experimental setup

The FDML laser consists of a silicon photonic integrated MDR, an erbium-doped fiber amplifier (EDFA), a 9-km long none-zero dispersion-shifted single mode fiber (NZDS-SMF) and a wave-shaper (WS). The photonic integrated MDR plays a key role in the system to perform tunable and narrow-band filtering required for Fourier-domain mode locking. In the experiment, the MDR is thermally tuned by using an electrical signal from a function generator, to select an optical mode at a time. To compensate for the loss in the loop, an EDFA as a gain medium is employed in the laser cavity to provide a sufficiently large gain over the swept wavelength range. The NZDS-SMF with a length of 9 km in the laser cavity provides an optical delay of around 44.7 µs. Thus, the repetition time of the driving signal applied to the MDR is around 44.7 µs, which is slow enough for the MDR to respond to. The role of the WS is twofold: first, it acts as a wideband optical filter with a bandwidth of 10 nm to filter out the undesired amplified spontaneous emission (ASE) noise from the EDFA, and second, it is programmed to partially compensate for the dispersion in the laser cavity to make the loop dispersion free. An isolator is also incorporated in the laser cavity, which is used to ensure unidirectional operation of the FDML laser. An optical coupler (OC1) with a coupling ratio of 10:90 is also employed to couple out 90% power (6.8 dBm) from the laser loop for the heterodyne beating. A narrow linewidth (<1 kHz) optical carrier from a tunable LD with an output power of 0 dBm is combined with the generated frequency-chirped optical pulse at a second optical coupler (OC2) and applied to a high-speed PD (50 GHz) to generate a LCMW. Finally, the generated LCMW is recorded by a real-time digital oscilloscope with a sampling rate of 160 GSa/s.

### MDR design and layout

To increase the Q factor, the MDR is designed to have an additional slab layer with a thickness of 90 nm to wrap the disk and the bus waveguides (shown in Fig. 1a and b). The radius of the disk is 10 µm and the thickness is 220 nm. In order to effectively excite the first-order radial TE mode supported by the MDR, the width of the bus waveguide is selected to 600 nm to meet the phase-matching condition. The gap width between the disk and bus waveguide is set at 200 nm to meet the critical coupling condition. For simplicity in testing, four arrayed TE-mode grating couplers that are spaced with a spacing of 127 nm are used to couple light between an arrayed fiber coupler and the MDR chip. Between the grating coupler and the MDR, four strip waveguides are used to guide the optical signal. Since an additional slab layer is added to wrap the bus waveguide of the MDR, a linear taper slab waveguide with a length of 25 μm is used for mode transition between the strip and bus waveguides. In order to further stabilize the filter response of the MDR, the silicon chip is placed on a temperature-controlled platform to stabilize the temperature at 23 °C when the MDR is not tuned.

## Supplementary information

Supplementary Info

## Data Availability

The data that support the findings of this study are available from the corresponding author upon request.
