# Supplementary material for: Hybrid Fourier-domain mode-locked laser for ultra-wideband linearly chirped microwave waveform generation
Source: Nat Commun. 2020 Jul 30;11:3814. doi: 10.1038/s41467-020-17264-8 (PMC7393121; doi:10.1038/s41467-020-17264-8)
Supplement: Supplementary file 1 — Supplementary Info [file 41467_2020_17264_MOESM1_ESM.pdf]

Supplementary Information for
Hybrid Fourier-domain mode-locked laser for ultra-wideband linearly chirped microwave waveform generation

Jian Tang et al

Supplementary Note 1: The performance of the FDML laser

The performance of the Fourier-domain mode-locked (FDML) laser depends largely on the electrical signal applied to the micro-disk resonator (MDR) in the laser cavity. Here, the optical spectrums and the corresponding temporal profiles of the frequency-chirped optical pulses from the FDML laser under different peak-to-peak (V_{PP}) voltages applied to the MDR are measured. During the measurement, a driving signal with a repetition time of 44.7 μs having a parabolic profile is applied to the micro-heater on the MDR. The optical spectrum of the frequency-chirped optical pulse is measured by an optical spectrum analyzer (OSA). As can be seen, the wavelength swept range is broadened from 0.08 nm (10 GHz) to 0.80 nm (100 GHz) when the V_{PP} applied to the MDR is increased from 350 mV to 1040 mV (shown in Supplementary Fig. 1a, b, c and d). This is because a higher V_{PP} of the driving signal has a higher electrical power, which makes the spectral response of the MDR have a wider bandwidth. Thus, a broader wavelength swept range could be expected by applying a higher V_{PP} to the MDR. However, a too high voltage applied to the MDR may damage the thermal resistance in the MDR. Thus, the frequency swept range of the FDML laser is limited by the highest V_{PP}. Supplementary Fig. 1e, f, g and h shows the temporal profiles of the frequency-chirped optical pulses after the photodetection at a high-speed photodetector (PD), captured by a real-time oscilloscope. The temporal duration of the frequency-chirped optical pulses is fixed at 30 μs with the increase in the V_{PP}. While the intensity profiles are degraded with the increase of the V_{PP}. When the V_{PP} is increased to 1040 mV, the temporal profiles become noisy with large ripples, as shown in Supplementary Fig. 1h. This is caused by the increase in the dispersion since the wavelength swept range is increased, which leads to increased time delay errors, making Fourier-domain mode locking less effective. Thus, the performance of the FDML laser when operating at a higher V_{PP} is poorer. A solution is to compensate the dispersion in the laser cavity over the entire wavelength swept

range to ensure an identical round trip time over the entire bandwidth.

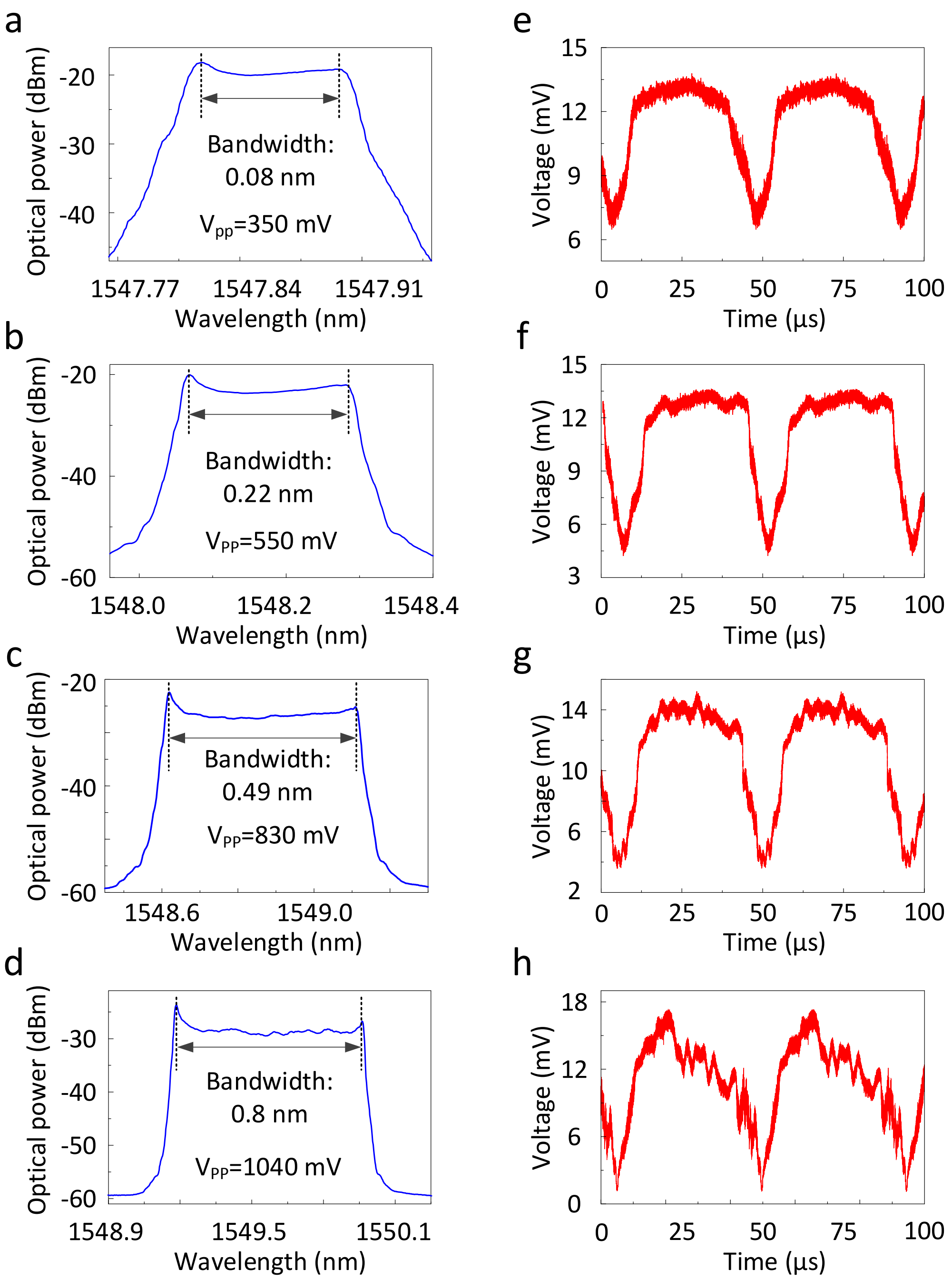

Supplementary Fig. 1 The optical spectrums of the frequency-chirped optical pulse from the FDML laser when the V_{PP} of the driving signal applied to the micro-heater on the MDR at **a.** 350 mV, **b.** 550 mV, **c.** 830 mV and **d.** 1040 mV. The corresponding temporal profiles of the optical chirped pulses at the output of the FDML laser when the V_{PP} is applied to the micro-heater on the MDR at **e.** 350 mV, **f.** 550 mV, **g.** 830 mV and **h.** 1040 mV.

Supplementary Note 2: The temporal profile of the driving signal

The chirp linearity of the generated LCMW is dependent directly on the chirp linearity of the frequency-chirped optical pulse, which is determined by the frequency tuning response of the MDR. To ensure a linear frequency tuning response of the MDR, the time-domain profile of the driving signal shoud have a function given by $U(t) = 1 - A/(\sqrt{t} + B)$, where, t is the time, A is the amplitude factor and B is the shape correction factor. In the experiment, the time-domain profile of the driving signal is shown in Supplementary Fig. 2, in which A is set at 1 and B is set at 0.00085. The parameters of the generated LCMW, in terms of bandwidth, duration and reputation rate, can be tuned by tuning the peak-to-peak voltage (Vpp), temporal duration and repetition time of the driving signal, respectively. The driving signal is generated by a function generator (Agilent 33250A) in the experiment.

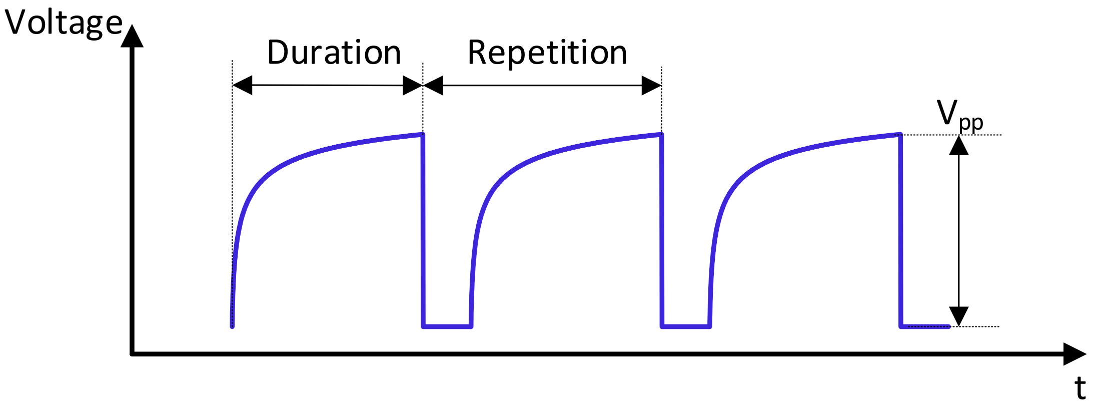

Supplementary Fig. 2 The time-domain profile of the driving signal.

Supplementary Note 3: Characterization of the linearity of the generated LCMW

To evaluate the chirp linearity of the generated LCMW, linear fitting of the instantaneous frequency of the generated LCMW is performed. The linearity is quantitatively evaluated by its R-square value. For a generated LCMW with a time duration of 30 μs and a bandwidth of 50 GHz, the R-square value is calculated to be0.99763, which is very close to 1, indicating a

good linearity of the generated LCMW. The instantaneous frequency of the generated LCMW and its linear fitting is shown in Supplementary Fig. 3.

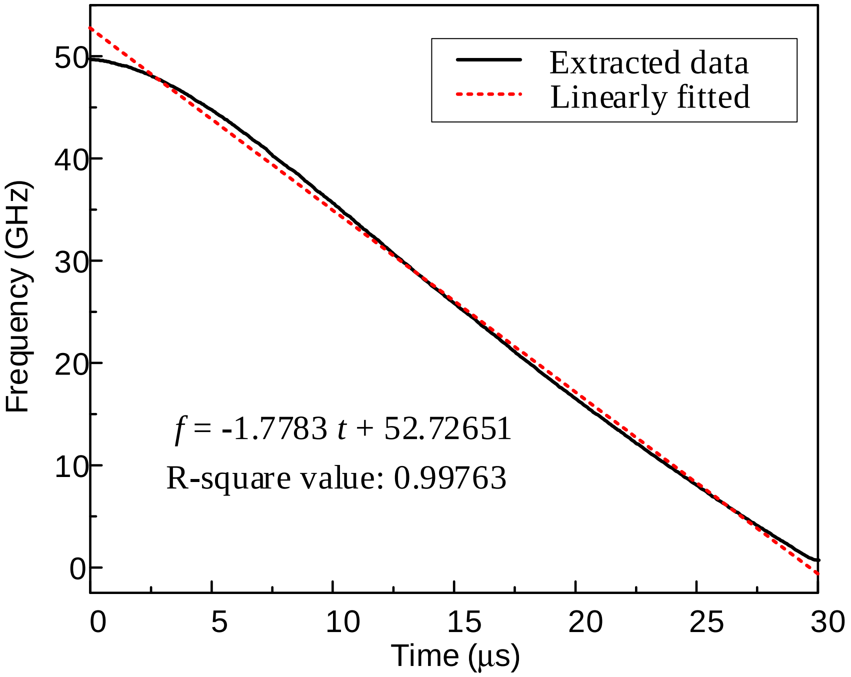

Supplementary Fig. 3 The instantaneous frequency of a generated LCMW and its linear fitting.
